# Production of fluorescent dissolved organic matter in Arctic Ocean sediments

**DOI:** 10.1038/srep39213

**Published:** 2016-12-16

**Authors:** Meilian Chen, Ji-Hoon Kim, Seung-Il Nam, Frank Niessen, Wei-Li Hong, Moo-Hee Kang, Jin Hur

**Affiliations:** 1 Department of Environment & Energy, Sejong University, Seoul 05006, South Korea; 2 Petroleum & Marine Division, Korea Institute of Geoscience and Mineral Resources, 124 Gwahang-no, Yuseong-gu, Daejeon 34132, South Korea; 3 Division of Polar Paleoenvironment Korea Polar Research Institute, Incheon 21990, South Korea; 4 Alfred Wegener Institute, Helmholtz Centre for Polar and Marine Research (AWI), Am Alten Hafen 26, 27568 Bremerhaven, Germany; 5 CAGE - Centre for Arctic Gas Hydrate, Environment and Climate, Department of Geology, UiT The Arctic University of Norway, Tromsø, Norway

## Abstract

Little is known about the production of fluorescent dissolved organic matter (FDOM) in the anoxic oceanic sediments. In this study, sediment pore waters were sampled from four different sites in the Chukchi-East Siberian Seas area to examine the bulk dissolved organic carbon (DOC) and their optical properties. The production of FDOM, coupled with the increase of nutrients, was observed above the sulfate-methane-transition-zone (SMTZ). The presence of FDOM was concurrent with sulfate reduction and increased alkalinity (R^2^ > 0.96, *p* < 0.0001), suggesting a link to organic matter degradation. This inference was supported by the positive correlation (R^2^ > 0.95, *p* < 0.0001) between the net production of FDOM and the modeled degradation rates of particulate organic carbon sulfate reduction. The production of FDOM was more pronounced in a shallow shelf site S1 with a total net production ranging from 17.9 to 62.3 RU for different FDOM components above the SMTZ depth of *ca.* 4.1 mbsf, which presumably underwent more accumulation of particulate organic matter than the other three deeper sites. The sediments were generally found to be the sources of CDOM and FDOM to the overlying water column, unearthing a channel of generally bio-refractory and pre-aged DOM to the oceans.

The Arctic Ocean is intimately associated with global carbon and hydrological cycling as well as climate change. A large stock of organic carbon (1100–1500 Pg C), including permafrost, is contained in its drainage basin area, which is sensitive to climate change^1^. The Arctic Ocean also influences formation of North Atlantic Deep Water which drives Atlantic thermohaline circulation. The fluvial discharge into the Arctic Ocean is reported to be 25–36 Tg C yr^−1^ for dissolved organic carbon (DOC) and around 12 Tg C yr^−1^ for particulate organic carbon (POC)^1^,^2^,^3^. It was estimated that roughly over 80–90% of the organic carbon, carried by fluvial runoff, is buried in shallow coastal sediments^4^. Despite the large fraction of the organic carbon contained in the global oceanic sediments (*ca.* 700 Pg C)^5^, much is unknown about the dynamics of sediment pore water dissolved organic matter (PW-DOM). It was previously reported that sediment oxygen consumption rates were much faster in coastal sediments ( > 200 mM O_2_ m^−2^d^−1^) than in deep sea sediments (*ca.* 0.02 mM O_2_ m^−2^d^−1^)^6^, implying a higher biological respiration occurring in coastal sediments. The huge difference of more than four orders of magnitude of oxygen consumption rates may reflect the huge differences of organic matter supply. With the prediction of ice-free summer in the Arctic Ocean over the next few decades^7^, it is urgent to understand the dynamics of sediment DOM in this area as increasing organic carbon loading via fluvial runoff can be expected with global warming underway. Whether the organic carbon burial rates will go up or down, however, is unknown at present.

Although the origins of sediment organic matter are diverse including riverine runoff, coastal erosion, sea ice, aeolian input, and autochthonous primary productivity in the Arctic^8^, a substantial fraction is known to be of terrigenous origin^9^,^10^,^11^,^12^. While solar irradiation is usually the major sink for terrigenous chromophoric DOM (CDOM), autochthonous organic matter produced by surface primary productivity is generally considered susceptible to microbial utilization^13^,^14^,^15^. Meanwhile, the majority (*ca.* 80%) of the organic matter sinking to seafloor is channeled into benthic respiration^16^. Once settling into sediments, the remainder organic matter undergoes a complex combination of physical (e.g., adsorption), chemical (e.g., redox reactions), and biological (e.g., aerobic and anaerobic respiration) diagenetic processes. The ultimate preservation rate is estimated to be <0.5% of the gross production of primary productivity^17^,^18^,^19^.

Although there have been a number of DOM studies on Arctic Ocean seawaters and arctic rivers^12^,^20^,^21^,^22^,^23^,^24^,^25^,^26^, little attention has been paid to Arctic sediment. A recent report on PW-DOM from surficial sediments in the Eurasian Basin has shown the relationships between the molecular signature and sea-ice coverage, which illustrated a higher abundance of peptide and aliphatic molecular formula in productive ice margins as compared to the molecular features (e.g., more aromatic) found in multiyear ice-covered stations^27^. Other related studies primarily used biomarkers or stable isotopes simply to distinguish between terrigenous and marine sources and found a significant fraction of terrigenous organic matter in the Arctic Ocean^9^,^10^,^11^,^28^,^29^,^30^. Studies of Arctic PW-DOM are essential in exploring the DOM characteristics and dynamics in the sediments as well as potential interactions with the overlying water column.

Optically active DOM is widely used to trace the dynamics of DOM in aquatic environments. These DOM pools (i.e., CDOM and FDOM) from terrestrial-derived humic substances in surface waters are generally observed to be bio-refractory while some fresh biomass-derived fractions could be readily utilized^13^,^14^. The production of FDOM in oxic or suboxic ocean interior was previously reported, which was explained by biological oxidation of sinking particulate organic matter (POM) based on a positive relationship between FDOM and AOU (apparent oxygen utilization)^31^. These previous findings drive the investigation of CDOM and FDOM in oceanic sediment pore waters with a hope that it could provide valuable information regarding organic carbon storage and the potential benthic efflux into the ocean water column. Investigation of this potential production and efflux of FDOM in oceanic sediments can help to reconcile the reactivity of dissolved organic matter and its age in the deep oceans. Excitation-emission matrix coupled with parallel factor analysis (EEM-PARAFAC) is an ideal method for FDOM studies because it can provide quantitative information on decomposed fluorescent components from three-dimensional fluorescence spectra of DOM^32^. The optical method has proven successfully utilized in examining PW-DOM in both oceanic and freshwater ecosystems^14^,^33^,^34^,^35^,^36^.

The objectives of this study were (1) to characterize the Arctic sediment PW-DOM with different depths (up to 10.5 meters below seafloor (mbsf)) in the Chukchi-East Siberia Sea area along a shelf-slope-basin gradient, (2) to infer the potential origins, diagenetic transformations, and the paleoenvironments associated with the DOM variations, and (3) to explore the potential production of FDOM in the sediments and benthic flux of DOM in the water-sediment interface. The study area is of interest for this topic in that (1) there is a seasonal sea ice cover in the region so it is very sensitive to climate change, (2) it is currently affected by nutrient-rich Pacific water inflow through the Bering Strait so it is easily affected by high primary productivity, which is known to be an important source of organic matter in this region, and (3) the marginal seas are characterized by high sedimentation rates. Sediment cores roughly reflect the geological time spanning up to mid- to late Quaternary deposit based on the age-depth models at sites in close proximity^37^,^38^. The major notable paleoenvironmental events occurring during the geological period include late Quaternary glacial/interglacial cycles^36^ and the reopening of the Bering Strait during the Early Holocene at *ca.* 10.5 ka^39^,^40^. The opening of the Bering Strait is expected to deliver more nutrients and thus promote the primary productivity in this region.

## Results

### Downcore profiles of water chemistry, DOC, CDOM, and FDOM

**Figure 1:**
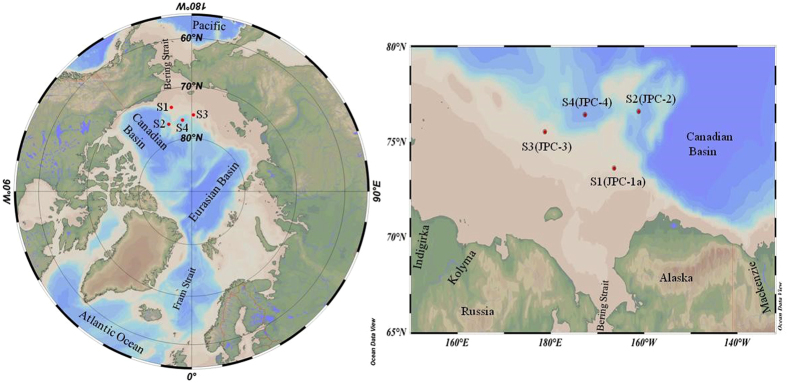
Sampling sites for sediment pore waters in the Chukchi Shelf (JPC-1a or S1), Northwind Basin (JPC-2 or S2), East Siberian continental slope (JPC-3 or S3), and Chukchi Basin (JPC-4 or S4) of the Arctic Ocean. The sea ice concentration data from August 27^th^ to September 5^th^, 2015 were obtained from http://www.meereisportal.de (grant: REKLIM-2013-04) (Refer to Spreen *et al*.^70^). The map was created by using Ocean Data View^71^.

The cores from sites S1, S2, S3, and S4 are 6.84, 3.54, 4.92, and 10.50 mbsf, respectively (Fig. 1). The high resolution sub-bottom profiles (SBP) of the sites are shown in the supporting information (Fig. S1). As seen from the SBP, there is no seismic vent at the study sites. The salinity averaged *ca.* 33.7 to 36.8 psu among the four sites (Tables S1 and S6). A sharp increase of alkalinity in contrast to a sharp decrease of sulfate was observed at shelf site S1. Alkalinity at site S1 increased from seafloor at 2.6 mM to 4.3 mbsf at 43.3 mM, and it remained a relatively constant below the depth in contrast with the sulfate concentration decreasing from the seafloor at 26.0 mM to about 4.1 mbsf at 0.0 mM (Fig. S2). The alkalinity is generated and sulfate is consumed via particulate organic matter sulfate reduction (POCSR: 2CH_2_O + SO_4_^2−^ → H_2_S + 2HCO_3_^−^) and anaerobic oxidation of methane (AOM: CH_4_ + SO_4_^2−^ → HS^−^ + HCO_3_^−^ + H_2_O) reactions in the sediments. The rapid changes of both alkalinity and sulfate signify the fast POCSR and AOM reactions which might be due to large amount of decomposable organic matter inputs to the shallow shelf site S1. The sulfate downcore profiles revealed that the sulfate-methane-transition-zone (SMTZ) is about 4.1 mbsf at site S1, while the other sites had not reached SMTZ yet up to the studied depths (Table S1).

**Figure 2:**
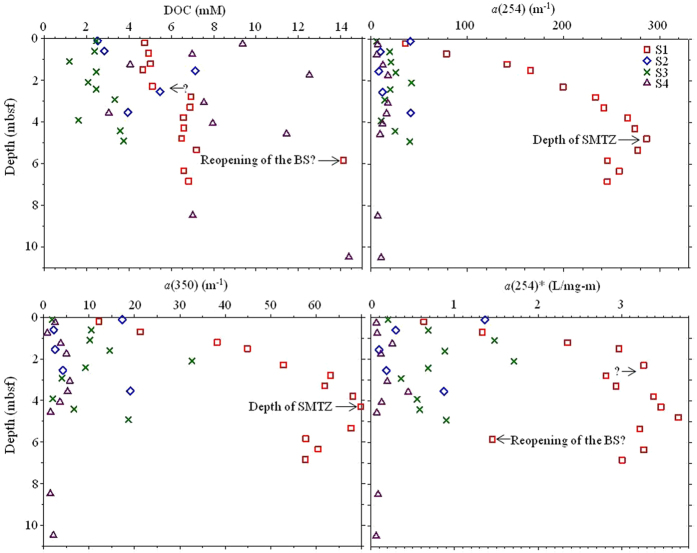
Downcore profiles of the sediment pore water in the Arctic (BS: Bering Strait).

The bulk DOC data for pore waters at sites S1, S2, S3, and S4 exhibited the average concentrations of 6.6 ± 2.4, 4.4 ± 1.9, 2.5 ± 0.8, and 8.4 ± 3.6 mM, respectively. These DOC values were 1–2 orders of magnitude higher than those reported for the surficial sediments (0–10 cm) from the Amundsen and Nansen Basins located in the Eurasian Basin (0.04–0.35 mM)^27^. For this study, the DOC values at the similar surficial sediments with the depths of 0–10 cm were even on the same order of magnitude as those of the deeper cores (Fig. 2). Thus, a much higher DOC appears to be stored in this study area relative to the Amundsen and Nansen Basins probably due to the high *in situ* primary productivity (up to 400 g C m^−2^ yr^−1^)^41^ and/or the high input of terrestrial organic matter. The DOC depth profiles were highly variable among the sites and the core depth, implying the interplay of complex factors (e.g., sea-ice coverage, seasonality of riverine runoff, ice-rafted debris sediments, nutrients availability, switch of ocean currents, reopening of the Bering Strait) during the late Quaternary glacial/interglacial cycles. The bulk DOC may be more affected by the above mentioned factors because it encompasses a broader DOM pool than CDOM and FDOM, and thus its reactivity range should also be broader. In contrast, the CDOM, expressed as absorption coefficient *a*(254) and *a*(350), displayed more obvious trends, especially for site S1, with a general increase followed by a gradual decrease with the core depth. Meanwhile, an apparent inflection point was observed at the shelf site S1, which coincides with the SMTZ depth (*ca.* 4.1 mbsf) inferred from the sulfate profile.

**Figure 3:**
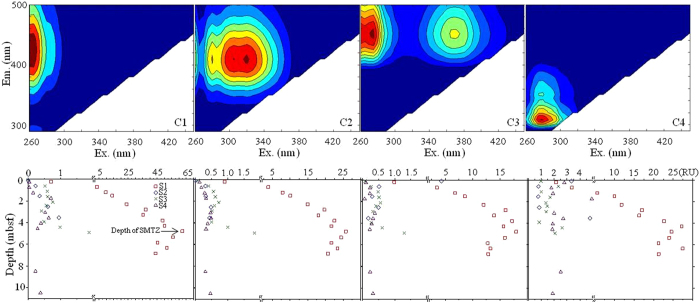
Contour plots of four identified EEM-PARAFAC components (upper panel) and downcore profile of the absolute abundance (RU, lower panel).

In this study, four different fluorescent components (C1–C4) were identified from the EEM-PARAFAC modeling (Fig. 3 and Fig. S3). From the spectral features, C1 (Ex/Em maxima: 265/422 nm) was assigned as a traditionally termed terrestrial humic-like component with Ex maximum in an ultraviolet range. C2 (Ex/Em maxima: 318/410 nm), C3 (Ex/Em maxima: 275(370)/452 nm), and C4 (Ex/Em maxima: 280/308 nm) were referred to marine/microbial humic-like, terrestrial humic-like, and protein-like components, respectively^13^,^14^,^32^. Similar to CDOM, the fluorescent components at site S1 showed an increasing trend with depth down to an obvious turning point around the SMTZ depth (*ca.* 4.1 mbsf) followed by a gradual decrease to the deeper sediments (Fig. 3).

### Variations of DOM parameters along the shelf-slope-basin gradient

The average bulk DOC values were 6.6 ± 2.4 mM at the Chukchi Shelf site S1, which may be explained that the shallower shelf area usually traps more terrestrial organic matter, resulting in a higher organic carbon burial^4^,^8^. At the Chukchi Basin site S4, the average DOC values were 8.4 ± 3.6 mM. Since site S4 is a basin site, the main DOM sources could be associated with *in situ* marine phytoplankton productivity. This explanation is supported by the low aromaticity proxy (i.e., *a*(254)*) at the site. The possible causes for the high phytoplankton productivity in this area will be discussed in the following section. The East Siberia Continental Shelf site S3 demonstrated a relatively lower DOC level (2.5 ± 0.8 mM, *p* < 0.01) among the four studied sites, implying that other factors such as autochthonous production, sea ice cover, and ocean currents rather than riverine runoff could be dominantly operative for the carbon preservation. It is notable that even the labile phytoplankton-derived DOM could be preserved in sediments in the case of a fast sedimentation (typical in inner shelf), in which the fresh organic materials could escape the respiration and preserved in the sediments^4^,^11^. For the CDOM, shelf site S1 showed much higher absorption coefficients (*p* < 0.0001) of *a*(254) (211 ± 78 m^−1^) and *a*(350) (53 ± 18 m^−1^), as compared to the other sites that displayed an order of magnitude lower values of *a*(254) (12–23 m^−1^) and *a*(350) (3–9 m^−1^). These results are interesting in that this site is relatively remote from a large river discharge. Similar to the CDOM patterns, the absolute abundances of the fluorescent components showed the highest level at site S1 (*p* < 0.0001).

### Correlations among DOM variables and water chemistry parameters

**Figure 4:**
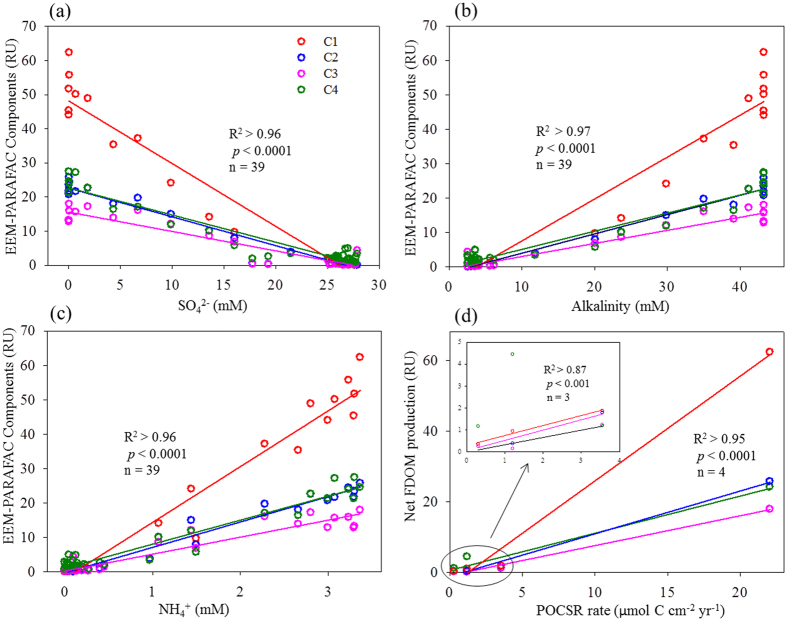
Inverse correlation between EEM-PARAFAC components and sulfate (**a**) concurrent with positive correlation with alkalinity (**b**) and ammonium (**c**) in Arctic pore waters. Positive correlations between net FDOM production and modeled POCSR reaction rates (**d**). The inset in (**d**) indicates the correlations for the clustered data.

Water chemistry could affect the optical properties of DOM^42^,^43^. To assess the influence, Pearson’s correlations were performed among the DOM variables and measured water chemistry parameters (Table S3). No significant correlations were found between DOC and CDOM or FDOM, suggesting either the uncoupled production mechanisms of DOC vs. optically active DOM or different reactivity between bulk DOC and CDOM or FDOM. Instead, strong positive correlations (R^2^ > 0.95, *p* < 0.0001) were observed for either CDOM or FDOM with nutrients (i.e., NH_4_^+^ and PO_4_^3−^, Figs S4–S5), suggesting the existence of the potential coupling processes to regulate CDOM, FDOM, and nutrient cycling in the studied sediments. The trends were in line with the positive correlations of FDOM with alkalinity, and the negative correlations with sulfate (R^2^ > 0.96, *p* < 0.0001, n = 39, Fig. 4a,b, Table S3).

## Discussion

The origins of PW-DOM in the study area could be diverse from riverine runoff, coastal erosion, aeolian inputs, and ice-rafted debris to autochthonous production. The riverine inputs have a strong seasonality although they occur mainly during spring freshet in modern conditions^8^. The relative strength of each source likely depends on specific depositional times and locations, and subsequent diagenetic processes. The high variations of DOC observed in this area may be attributed to the Quaternary glacial/interglacial periods corresponding to the core depth^37^. Moreover, the Chukchi Borderland and the East-Siberian continental margin were glaciated by more than 1000 m-thick ice during several Quaternary glaciations prior to the Last Glacial Maximum (Marine Isotope Stage 2)^44^,^45^. Grounded ice can significantly contribute to reworking of older terrestrial and marine sediments. The sea ice coverage regimes are completely different during the glacial versus the interglacial periods, affecting the entire hydrology and primary productivity.

The optical indices of humification index (HIX), biological index (BIX), fluorescence index (FI), specific absorption coefficient *a*(254)*, and slope ratio (S_R_) were utilized here to trace the origins and characteristics of the optically active DOM. Detailed definitions and descriptions of these proxies are found elsewhere^46^,^47^,^48^,^49^,^50^. The FI (>1.7) and HIX (<6) suggested a primarily microbial/marine-derived CDOM and FDOM origins with the exception of site S2 (Fig. 2 and Fig. S6). The bulk elemental results of C_org_/N_org_ were reported to be generally <10 in the Chukchi Sea region^38^, consistent with the optical data of this study. The Northwind Basin site S2 seems to reflect the optical signals of potential terrigenous DOM inputs of fulvic acids with relatively low FI (<1.5). This site is close enough to the Canadian Basin to be affected by the discharge of Mackenzie River through the Beaufort Gyre. Episodic ice-rafted debris inputs as well as reworking by grounded ice on the Northwind Ridge and Chukchi Plateau during glacial times could also be the explanations for the exceptional results^45^. However, aromaticity index of *a*(254)* was relatively low (0.6 ± 0.5 L(mgC-m)^−1^) and the protein-like C4 (could be a mixture of proteinaceous materials and phenolic moieties)^51^ was high (2.1 ± 1.9 RU), signifying its mainly marine/microbial-derived PW-DOM source. The highest average DOC values observed at site S4 can be ascribed to the lateral transport of both nutrients and DOM from the Chukchi Shelf to the Chukchi Basin^52^, which may stimulate the primary productivity followed by the increase of freshly produced biomass. This explanation is well supported by our results of the extremely low aromaticity index *a*(254)* (0.1 ± 0.1 L(mgC-m)^−1^) and the high protein-like C4% (72 ± 9%) at site S4. Although the potential linkage of the protein-like component with phenolic compounds was previously reported^51^, the possibility can be excluded here because of the extremely low aromaticity index *a*(254)*. Isotopic and biomarker analyses indicated a significant fraction of terrigenous organic matter in the Arctic Ocean^9^,^10^,^28^. However, our results suggest that spatial variations should be taken into account as an important factor. For the areas far away from direct large riverine influences, the DOM sources could be primarily microbial/marine-derived. A study using polyunsaturated fatty acids and algal sterols has reported relatively unaltered marine-derived organic matter in the Chukchi shelf and slope area^11^. An organic carbon to nitrogen ratio (i.e., C_org_/N_org_) of <10 at nearby sites also supports the dominant source of algal-derived organic matter in the Chukchi Shelf 29,53.

Within the oceanic sediment column, sulfates from overlying seawater or from re-oxidized sulfides operate as important electron acceptors above the SMTZ of sediments (typically about 1 to 100 mbsf), and they are involved in the two chemical reactions of POCSR and AOM. The POCSR potentially produces DOM. POCSR was reported to dominate organic matter degradation above SMTZ in sediments without seismic vents or faults^54^. The study sites here did not display seismic vents in the sub-bottom profiler (Fig. S1). As such, it is reasonable to assume POCSR as the dominant pathway of organic matter degradation at the sediments above SMTZ for this study. This is the first report on the production of FDOM in the Arctic sediments. The net increases (=the value at the indicated depth minus the value at the bottom water) of all the fluorescent components were found at all the studied sites, which are summarized in Table 1. The net increases of four EEM-PARAFAC components (C1 to C4) of 62.3, 25.7, 17.9, and 24.1 RU, respectively, were observed for the depth up to the SMTZ at site S1 (*ca.* 4.1 mbsf). For the studied depths at the sites S2, S3, and S4 (3.5 to 10.5 mbsf), the net increases ranged from 0.2 to 4.5 RU for all EEM-PARAFAC components. The FDOM increases with depth should not be due to the diffusive loss to the water column giving the huge differences of depth profiles between the shelf and basin sites. The quantity of the FDOM production was two orders of magnitude higher at site S1 than at the other sites. The shallower shelf site has a much shallower water depth of 100 m in comparison with the other slope and basin sites with much deeper water depths (S2: 2077 m; S3: 715 m; S4: 2240 m, Table S1). Furthermore, the C1 FDOM component at site S1 showed the highest production (62.3 RU) among all the fluorescent components and all the sites. This component has been reported to be a photo-refractory and/or photoproduct in a coastal wetland ecosystem^14^,^55^,^56^, and it was also found in a high abundance in canal water draining agricultural areas with “old” historic peat-derived DOM^57^. It is notable that the above-mentioned environments could be similar to the case of the “old” permafrost-derived organic matter immobilized from the surrounding continents via the coastal erosion and fluvial runoff to the Arctic sediments. Although DOM optical signatures were primarily marine/microbial-derived in this study area as discussed above, the biogenic particles may carry terrigenous organic matter, which could be transformed to DOM during diagenesis through biological oxidation and re-working.

In this study, the production of FDOM was accompanied by the increases of nutrients of ammonium and phosphate (i.e., NH_4_^+^ and PO_4_^3−^), concurrent with increased alkalinity and reduced sulfate (SO_4_^2−^, R^2^ > 0.96, *p* < 0.0001, n = 39, Fig. 4a–c, Figs S4–S5), suggesting the presence of a biological pathway for the production via anaerobic respiration rather than physical dissolution or fragmentation. If FDOM was produced simply by fragmentation or physical dissolution, such strong correlations with the consumption of sulfate, releases of nutrients, and increase of alkalinity would not be observed. Benefiting from the advantages of EEM-PARAFAC to decompose fluorescence EEMs into the four individual components, we found that the most dominant FDOM component produced was the terrestrial humic-like C1, known as photoproducts and/or photo-refractory substances, which is potentially associated with the permafrost-derived organic matter. Decreasing HIX with core depth, concomitant with the increases of BIX and S_R_ values, indicate increased biological activities and a lower molecular weight of CDOM with core depth (Fig. S6), supporting our inference on the origins of FDOM and the subsequent diagenesis in the sediments. Moreover, positive correlations between EEM-PARAFAC components and ammonium concentration and between net FDOM production and the modeled POCSR rates (see supporting information for the details of POCSR rates modeling) were observed (R^2^ > 0.95, *p* < 0.0001, n = 4, Fig. 4d), corroborating the FDOM production via anaerobic degradation of particulate organic matter. Although n is only four here, the correlation is strong as seen from the high R^2^ (>0.95) as well as significant test value of *p* < 0.0001 for all four EEM-PARAFAC components C1–C4. The correlation remains strong (R^2^ > 0.87, *p* < 0.001, n = 3) for most of the components (C1 and C3) at the relatively clustered sites S2, S3, and S4. Our observation is also in line with a previous report on the assimilation of particulate organic carbon by archaea above SMTZ in sediments^58^. As the rates of POCSR were simulated with two extreme scenarios of input parameters (i.e., the highest and lowest estimations, see supporting information), we expect that the potential uncertainties involved in POCSR rates modeling would be marginal.

The apparent production of FDOM above the SMTZ in the Arctic sediments was observed, which was associated with the organic matter degradation in the sediments as evidenced by the positive correlation with nutrients as well as the POCSR rates, and by the inverse correlation with sulfates. The subsequent flux estimation based on the Fick’s first law of diffusion (Diffusion flux J = sediment porosity at the water-sediment interface × diffusion coefficient × concentration gradient, i.e., J = ɸ_o_Ds(əC/əz)_o_) showed that most of the sites served as the sources of CDOM and FDOM (primarily for shelf site S1) to the overlying water column (see Supporting Information, Table S5). The continental shelf receiving a large terrestrial organic matter loading seems to be a hotspot of FDOM production in the sediments and subsequent efflux to the water column. In contrast, the slope and basin sites S2, S3, and S4 illustrated much lower FDOM production and therefore lower FDOM benthic flux. Some exceptions did occur with potential influx of the protein-like component, presumably due to autumn phytoplankton bloom in the water column. Our results provide further insight into the refractory nature of deep oceanic DOM, partially explaining the discrepancy between the average age of 4,000–6,000 yrs of oceanic DOM and the oceanic mixing time of about 1,000 yrs, in that humic-like FDOM are generally bio-refractory in nature^13^,^14^. The continental shelf appears to be an important area for the benthic efflux of FDOM. Since the Arctic Ocean has the widest continental margin, occupying about one third of the global ocean shelf areas, our major findings including the production of FDOM, and the benthic fluxes of FDOM, are worthy of being treated as important factors in constructing conceptual models of global carbon cycling. In this study, EEM-PARAFAC revealed the potential co-existence of the efflux and the influx of different DOM moieties in Arctic sediments. In addition, there was high variability of DOM quantity and quality with core depth and among different sites.

## Methods

### Site description

Sampling sites are located in the shallow Chukchi Shelf (site ARA06C-JPC-1a), Northwind Basin (site ARA06C-JPC-2), East Siberia Continental Slope (site ARA06C-JPC-3), and Chukchi Basin (site ARA06C-JPC-4) of the western Arctic Ocean (Fig. 1). For simplicity, the four sampling sites are denoted here as the sites S1, S2, S3, and S4, respectively. The locations and the site description are summarized in Table S1. The sub-bottom profiler (SBP) data (see supporting information and Fig. S1) showed that the sediments are Holocene marine transgressive sediments (S1), hemi-pelagic sediments (S3), and mostly fine-grained homogeneous pelagic sediment (S2 and S4). The modern Chukchi Sea is well known as seasonally ice-covered and having an expansive shallow continental shelf and extremely high primary productivity^41^ driven by nutrient-enriched Bering Shelf Anadyr Water entraining through the Bering Strait. The Northwind Basin is separated from the Canadian Basin by the Northwind Ridge, in which Pacific Summer Water (PSW) was reported to cause the disproportionally large recent ice retreat in this area^59^. The East Siberia Sea is supposed to receive a larger riverine discharge as compared to the Chukchi Sea, due to two large Siberian rivers (i.e., Kolyma and Indigirka). The Chukchi Sea circulation is dominated by the Bering Sea Water (BSW, >80% of inflow, about 0.7 Sv) and the Alaskan Coastal Water (ACW)^60^. The BSW is a mixture of Bering Shelf Anadyr Water (BSAW) enriched with nutrients (NO_3_^−^ ≥ 20 μM) and Bering Shelf Water with a low level of nutrients (NO_3_^−^ ≤ 1 μM)^60^.

A stable stratification is generally maintained in Arctic seawater due to a large riverine freshwater discharge and sea ice melting^61^. Water masses usually exist in several layers, including the low-salinity surface mixed layer, a complex halocline intermediate water (50–200 m, Pacific-derived on the Bering Strait side while Atlantic-derived on the Fram Strait side), a warm and saline Atlantic Deep Water, and a layer of bottom water (>900 m)^8^,^62^. The water residence times (WRTs) for seawaters are variable depending on the layers. In general, relatively shorter (1 to10 yrs) WRTs were found for the surface and the halocline layers, while longer WRTs (25 to 30 yrs), for the Atlantic deep layer, and up to about 300 yrs for the bottom water^8^. Two major current systems (i.e., the Beaufort Gyre and the Transpolar Drift) dominate surface water circulation in the Arctic Ocean. The Beaufort Gyre occupies most of the Amerasian Basin, in which our study sites are located, while the Transpolar Drift is dominant in the Eurasian Basin. A switching between cyclonic and anticyclonic Beaufort Gyre was also observed^63^.

### Sampling

Sediment cores were collected at the sites using both Jumbo Piston Corer (JPC) and Box Corer from August to September 2015, during the RV ARAON Arctic Expedition (ARA06C). The surficial sediment porosity (Table S2) was calculated from Wet Bulk Density (WBD) determined by whole-core logging^64^ using the undisturbed box-cores (maximum length of 50 cm) to avoid the potential alteration of the surface sediments (usually centimeters) sampled by JPC. The overlying bottom water was carefully collected from undisturbed surface sediment cores. The pore waters in the sediment cores were slowly extracted by Rhizon samplers. The bottom and pore waters were both collected in acid-prewashed syringes and filtered through an in-line 0.20 μm disposable polytetrafluoroethylene filter. The Rhizon sampler was reported to extract relatively more humified FDOM, and lower DOC, CDOM, and protein-like FDOM as compared to the extraction based on centrifugation^35^. The aliquots were transferred into acid-prewashed plastic bottles (Nalgene^®^ high density polyethylene). The pore and bottom water samples for DOM analyses were both immediately stored in a freezer. The samples for the onboard analyses and ion measurements were stored in a refrigerator (about 4 °C).

### Analytical measurements

Salinity was determined by a Reflectometer, and alkalinity was analyzed by titration using 0.1 N HCl onboard. The reproducibility of alkalinity was <2% based on the repeated analyses of the IAPSO (i.e., International Association for the Physical Sciences of the Oceans) standard seawater. The PO_4_^3−^ and NH_4_^+^ were measured using colorimetric methods using a spectrophotometer (Shimadzu UV-2450) at 885 nm and 640 nm, respectively^65^. Details regarding the measurements of other ions are described elsewhere^66^. The sediment porosity was measured from box-cores. DOC concentrations of pore waters were measured using a total organic carbon analyzer (Shimazu TOC-VCPH) as non-purgeable organic carbon (NPOC) with an analytical reproducibility of <2%^67^. DOC of bottom water was not available due to limited sample volume. Absorption spectra were scanned from 240 to 800 nm on a Shimadzu 1800 ultraviolet-visible (UV-Vis) spectrophotometer (Shimadzu Inc., Japan). Fluorescence EEMs were obtained using a Hitachi F-7000 luminescence spectrometer (Hitachi Inc., Japan) at the excitation/emission (Ex/Em) wavelengths of 250–500/280–550 nm. The excitation and the emission scans were set at 5 nm and 1 nm steps, respectively. All samples were transferred into a special capped cuvette in a nitrogen-filled glove box for the UV-Vis and EEMs measurements. Further details for the EEM measurements and Raman Unit (RU) normalization are described elsewhere^68^. The procedures of post-acquisition corrections are available in a previous report^56^. PARAFAC modeling was performed using the MATLAB7.0.4 with the DOMFluor toolbox^69^. All the corrected pore water (n = 39) and bottom water (n = 4) EEMs were used for modeling. The number of fluorescent components was determined based on the split-half validation and an example of measured, modeled, and residual EEMs are shown in supporting information (Fig. S3).

## Additional Information

**How to cite this article**: Chen, M. *et al*. Production of fluorescent dissolved organic matter in Arctic Ocean sediments. *Sci. Rep.* **6**, 39213; doi: 10.1038/srep39213 (2016).

**Publisher's note:** Springer Nature remains neutral with regard to jurisdictional claims in published maps and institutional affiliations.

## Supplementary Material

Supplementary Information

## Figures and Tables

**Table 1:** Production of FDOM (unit: RU) in the Arctic Ocean sediments (^*^BW represents bottom water.

FDOM	S1			S2			S3			S4		
	BW*	4.1 mbsf**	Net increase	BW	3.5 mbsf	Net increase	BW	4.9 mbsf	Net increase	BW	10.5 mbsf	Net increase
C1	0.0	62.3	62.3	0.0	0.9	0.9	0.0	1.9	1.9	0.0	0.4	0.4
C2	0.0	25.7	25.7	0.0	0.4	0.4	0.0	1.8	1.8	0.1	0.5	0.4
C3	0.0	17.9	17.9	0.0	0.2	0.2	0.0	1.3	1.2	0.1	0.3	0.3
C4	0.4	24.5	24.1	0.3	4.8	4.5	0.9	2.1	1.2	0.7	1.9	1.2

^**^SMTZ depth. Net increase = value_indicated depth_ − value_BW_).
