# Supplementary material for: Production of fluorescent dissolved organic matter in Arctic Ocean sediments
Source: Sci Rep. 2016 Dec 16;6:39213. doi: 10.1038/srep39213 (PMC5159788; doi:10.1038/srep39213)
Supplement: Supplementary Information [file srep39213-s1.pdf]

Production of fluorescent dissolved organic matter in Arctic Ocean sediments

Meilian Chen[a], Ji-Hoon Kim[b], Seung-Il Nam[c], Frank Niessen[d], Wei-Li Hong[e], Moo-Hee Kang[b], and Jin Hur[a,*],

[a]*Department of Environment & Energy, Sejong University, Seoul 143-747, South Korea*

[b]*Petroleum and Marine Research Division, Korea Institute of Geoscience and Mineral Resources, 124 Gwahang-no, Yuseong-gu, Daejeon 34132, South Korea*

[c]*Arctic Research Centre, Korea Polar Research Institute, Incheon 21990, South Korea*

[d]*Alfred Wegener Institute, Helmholtz Centre for Polar and Marine Research (AWI), Am Alten Hafen 26, 27568 Bremerhaven, Germany*

[e]CAGE - Centre for Arctic Gas Hydrate, Environment and Climate, Department of Geology, UiT The Arctic University of Norway, Tromsø, Norway

Re-submitted to *Scientific Reports*, November, 2016

Corresponding Author:

*Phone: +82-2-3408-3826; fax: +82-2-3408-4320; e-mail: jinhur@sejong.ac.kr.

Supporting Information: Table S1-S6, Fig. S1-S7.

Sub-bottom profiler data: Sub-bottom profiler (SBP) data were collected during the ARA06C Expedition using Kongsberg SBP 120 system mounted on IBRV Araon. The frequency range of the system was 2.5 to 6.5 kHz. SBP data were recorded with a 1.5 sec ping interval and a 30 ms pulse length. Spiking filter and time variable gain (TVG) were applied to acquire the SBP signal, which enhances the resolution of sub-bottom profile image. Penetration depths of SBP data varied from few meters to hundreds meters below seafloor, depending on the physical properties of the sediments.

In the Chukchi Shelf (site S1), the acoustic basement showed grooved bed forms (Fig. S1), which resulted from iceberg keels ploughing through the sedimentary seabed during the last deglaciation[1-3]. Sediment in Unit S1-II on top of grooved acoustic basement infills and drapes the iceberg scours. Unit S1-I (~ 5 m), draping thin over Unit S1-II, is acoustically layered to transparent, which was identified as Holocene marine transgressive sediments[3].

In the Northwind Basin of the Arctic Ocean (site S2), reflectors in Unit S2-III showed sub-continuous to discontinuous layers with low amplitude. Unit S2-II, separated by a strong continuous reflector from the underlying Unit S2-III, is well stratified with low to medium amplitude reflectors. Thickness of Unit S2-II is uniform, corresponding to around 40 m. Acoustically well-layered thin seabed (~7 m) with medium to high amplitude draped the underlying Unit S2-II. Near the top, acoustically transparent sediment layers with low-medium amplitudes (Unit S2-I) are observed. Transparent layers pinch out near the slope, which may indicate gravity flows and lateral sediment transport, whereas intercalated stratified sediment with constant thickness indicates fine-grained homogeneous pelagic sediment.

In the East Siberia Continental slope (site S3), the Chirp-line resembles sub-bottom and seismic data previously published from the same location[4]. Acoustically continuous and well-stratified sediments (Unit S3-I) drape the transparent and unstratified underlying unit, which is considered glacial till based on the previous studies[3-6]. This hemi-pelagic drape had a uniform thickness of ~20 m, and it is divided into two sub-units (Unit S3-Ia and S3-I b) by a continuous high amplitude reflector. This reflector marks the stratigraphic position of a series of recessional moraines further up on the continental slope[4].

In the Chukchi Basin of the Arctic Ocean (site S4), acoustically continuous to sub-continuous reflectors with varying amplitude define Unit S4-III. In the upper part of Unit S4-III, wavy to semi-transparent sediment (Unit S4-II) with low-medium amplitude is developed. The Unit S4-III and S4-II were ~25 m to ~30 m in thickness. Well-laminated to transparent sediment (Unit S4-I) with medium amplitude reflectors drapes the S4-II Unit. This well-laminated to transparent unit (~ 20 m in thickness) consistently draping underlying sediment can be interpreted as fine-grained pelagic sediment like parts of Unit S2-I in Northwind Basin.

Surficial sediment porosity calculation: Sediment porosity (Fractional Porosity, FP) was calculated from Wet-Bulk Density (WBD) assuming constant grain densities and pore water densities (2.7 kg m^{-3} and 1.03 kg m^{-3}, respectively):

$$FP = (2.7\text{-}WBD) / (2.7\text{-}1.03)$$

WBD has been determined by whole-core logging using a Geotek (Ltd, UK) Multi-Sensor-Core-Logger and applying the gamma-ray attenuation method and calibration as described by Best and Gunn (1999)[7].

POCSR rates modeling: We constrained the rates of particulate organic carbon sulfate reduction (POCSR) by modeling pore water ammonium profiles with CrunchFlow, an approach that has been applied in numerous recent works[8-10]. Decomposition of organic matter by POCSR consumes sulfate and releases ammonium following the reaction:

$$(CH_2O)(NH_3)_{\frac{N}{C}}(H_3PO_4)_{\frac{P}{C}} + 0.5SO_4^{2-} \rightarrow 0.5HS^- + aHCO_3^{2-} + (\frac{N}{C})NH_4^+ + cCO_2 + eH_2O \quad (1)$$

The stoichiometry in Eq. (1) is determined by the TOC:N_{org} ratios of the organic matter[11]. The POCSR rate was formulated as *Monod-type* rate expression with one *Monod* term:

$$R_{POCSR} = A_m k_m \exp[\frac{-Ea}{RT}]\prod a_i^n [1 - \frac{Q}{K_{eq}}] \quad (2)$$

$$k_m = k_{\max}^{POCSR}\left(\frac{C_{SO4}}{C_{SO4} + K_{half}}\right) \quad (3)$$

where A_m (=1) and k_m are the surface area and kinetic constant. Ea, R, and T are the activation energy, ideal gas constant, and temperature. a_i^n is the activity product of solutes in the reaction with their stoichiometry (n) as exponents. $\frac{Q}{K_{eq}}$ determines the direction of reaction where Q is the ion activity product and K_{eq} is the equilibrium constant. As POCSR is a kinetic-driven reaction, we arbitrarily assigned a K_{eq} to ensure that the reaction always in a forward direction. $k_{\max}^{POCSR}$ is the theoretical maximum rate that was obtained by fitting the observed pore water ammonium profiles at all sites. K_{half} is the half saturation constant (=100 M)[12], and C_{SO4} is the concentration of sulfate.

Ammonium is also consumed through ion exchange on the surface of clay minerals, such as

smectite. The smectite content in this region is about 10-15% of the bulk sediments[13,14]. Assuming the cation exchange capacity (CEC) of smectite is 80-150 meq/100g of bulk sediments, we estimated the CEC for the sediments investigated is about 8 to 22.5 meq/100g of sediments. With these CEC values specified and chose the *Gaines-Thomas* activity convention in CrunchFlow, we can account for sink of ammonium in the sediments.

Other input parameters required for modeling are the age and porosity of the sediments. The age of the sediments at site S1 was estimated based on the age model constructed by Schade (2013)[11] for the site ARA2B1A-GC-02, which was estimated to be 9,194 cal. yr BP at 509 cmbsf (average sedimentation rate: ~55 cm ky^{-1}). The sedimentation rates were, however, an order of magnitude slower in the Chuckchi Basin (4.23 to 4.66 cm ky^{-1} for top 550 to 513 cmbsf sediments for the core PS72/340-5)[15]. We used the same age model by Schade (2013)[11] in a Chukchi Shelf site for all the sites and assumed constant down-core porosity with a minimum of 0.63 and a maximum of 0.86 in our simulations. This might cause the underestimation of the ages and overestimation of the POCSR rates at the slope and basin sites. Therefore, the estimation was rough since the sedimentation rates and age models are supposed to be different at each site. Due to the most pronounced FDOM production of site S1, we believe that the uncertainties of POCSR rates at other sites will have limited effects on the overall trends in this study. Only solute diffusion was considered in this simulation as fluid advection due to sediment burial and compaction will not result in rates that are significant compared to diffusion.

We simulated two scenarios with parameter combinations representing the highest and lowest rate estimations of POCSR for all four sites. Lower TOC to N_{org} ratios will result in more ammonium produced and therefore only requires a lower POCSR rate to fit the ammonium profile.

Higher CEC values represent greater ammonium consumption through cation exchange. Diffusion is faster when porosity is higher, and therefore more ammonium will escape from the sediments (i.e., less ammonium in the sediments). A faster POCSR is necessary to fit the pore water profile with faster diffusion. The POCSR rates derived from the modeling were integrated with depth for each site. The depth-integrated rates estimated under the different scenarios are summarized in Table S4.

Benthic flux of PW-DOM: The estimated benthic flux of CDOM and FDOM is summarized in Table S5, which was based on the Fick's first law of diffusion. DOC benthic flux was not estimated due to lack of bottom water DOC data. The estimation was rough since the diffusion interface is likely to be thin but the core depth data available here were from the surface to 20, 10, 10, and 25 cm for sites S1, S2, S3, and S4, respectively. For this study, an assumption of the linear concentration gradient in the upper 10, 20, or 25 cm sediments was made to partially compensate for the limited data. A diffusion-dominated system without a significant advection was also assumed for the estimation. The benthic flux was calculated with the depth-integrated average sediment porosity of 0.86, 0.67, 0.74, and 0.63 corresponding to the top sediments at sites S1, S2, S3, and S4, respectively, according to the porosity data calculated from box-cores, in which diffusion coefficients ranged from 0.8 to 1.9×10^{-6} $cm^{-2}yr^{-1}$ under a temperature of ~3 °C and the assumed average DOM molecular weight of ~1,000 Da[16]. The actual bottom water temperature was slightly lower than the assumed temperature (~ 0 °C), but the effect on the diffusion coefficient was supposed to be negligible. Our results showed that the majority of the sites (i.e., S1, S3, and S4) serve as the CDOM and FDOM sources to the overlying water column, which is in line with the current consensus[17]. The estimated efflux and influx trends only reflect the patterns at the

particular sampling time in late August and early September 2015 here. The shelf site S1 showed the greatest efflux for all the fluorescent components, ranging from 7.7 to 46.4 (RU L) $m^{-2}yr^{-1}$, while the highest efflux of CDOM was presented at site S2 ranging from 27.6 to 65.7 (m^{-1} L) $m^{-2}yr^{-1}$ (Table S5). Interestingly, site S2 showed the benthic efflux of the terrestrial humic-like C3 up to 16.0 (RU L) $m^{-2}yr^{-1}$. At sites S3 and S4, the influx of the protein-like C4 was 14.7 to 34.9 and 0.1 to 0.3 (RU L) $m^{-2}yr^{-1}$, respectively. Therefore, our study based on EEM-PARAFAC and CDOM revealed insights into the potential co-existence of the efflux and the influx of different DOM constitutes. The influx of the protein-like C4 at sites S3 and S4 might be associated with potential autumn algal bloom in the water column, which possibly took place in the sampling times (i.e., August 30th and September 2nd)[18,19]. The highest FDOM efflux in site S1 was very consistent with the high FDOM production (Table 1). However, since all estimations were based on one time measurements of the specific locations, further investigation based on more intensive sampling times and locations are required to obtain a better understanding of the DOM dynamics occurring in the Arctic Ocean sediments.

Non-metric multidimensional scaling (NMS) of the pore water samples: NMS was based on DOM optical data and water chemistry data. The ordination showed samples from site S1 were generally on the right side while those from sites S2 to S4 distributed on the left. From the loading plot, samples at shelf site S1 seemed more affected by CDOM, FDOM, and nutrients concentrations whereas those from the remainder sites were more dominated by salinity and protein-like component.

References

1. Hill, J. C. & Driscoll, N. W. Iceberg discharge to the Chukchi shelf during the Younger Dryas. *Quaternary Research* **74**, 57-62 (2010).

2. Polyak, L., Edwards, M. H., Coakley, B. J. & Jakobsson, M. Ice shelves in the Pleistocene Arctic Ocean inferred from glaciogenic deep-sea bedforms. *Nature* **410**, 453-457 (2001).

3. Dove, D., Polyak, L. & Coakley, B. Widespread, multi-source glacial erosion on the Chukchi margin, Arctic Ocean. *Quaternary Science Reviews* **92**, 112-122 (2014).

4. Niessen, F. *et al.* Repeated Pleistocene glaciation of the East Siberian continental margin. *Nature Geosci* **6**, 842-846 (2013).

5. Stoker, M. S., Stewart, F. S., Paul, M. A. & Long, D. in *Offshore Site Investigation and Foundation Behaviour: Papers presented at a conference organized by the Society for Underwater Technology and held in London, UK, September 22–24, 1992* (eds D. A. Ardus *et al.*) 239-262 (Springer Netherlands, 1993).

6. Ó Cofaigh, C. O. *et al.* Flow dynamics and till genesis associated with a marine-based Antarctic paleo-ice stream. *Quaternary Science Reviews* **24**, 709-740 (2005).

7. Best, A. I. & Gunn, D. E. Calibration of marine sediment core loggers for quantitative acoustic impedance studies. *Marine Geology* **160**, 137-146 (1999).

8. Hong, W.-L. *et al.* Removal of methane through hydrological, microbial, and geochemical processes in the shallow sediments of pockmarks along eastern Vestnesa Ridge (Svalbard). *Limnol Oceanogr* doi:10.1002/lno.10299 (2016).

9. Hong, W. L., Solomon, E. A., Torres, M. E. A kinetic-model approach to quantify the effect of mass transport deposits on pore water profiles in the Krishna–Godavari basin, Bay of Bengal, *Marine and Petroleum Geology* doi: 10.1016/j.marpetgeo.2014.06.014 (2014a).

10. Hong, W.-L., Torres, M. E., Kim, J.-H., Choi, J. & Bahk, J.-J. Towards quantifying the reaction network around the sulfate–methane-transition-zone in the Ulleung Basin, East Sea, with a kinetic modeling approach. *Geochim Cosmochim Acta* **140**, 127-141 (2014b).

11. Schade, I. M. *Holocene variability in Pacific-water inflow and primary productivity in the Chukchi Sea (Arctic Ocean): Reconstruction from biogenic sediment and physical property data* M.S. thesis, University of Bremen, (2013).

12. Wegener, G. & Boetius, A. An experimental study on short-term changes in the anaerobic oxidation of methane in response to varying methane and sulfate fluxes. *Biogeosciences* **6**, 867-876 (2009).

13. Wahsner, M. *et al.* Clay-mineral distribution in surface sediments of the Eurasian Arctic Ocean and continental margin as indicator for source areas and transport pathways — a synthesis. *Boreas* **28**, 215-233 (1999).

14. Ortiz, J. D. *et al.* Provenance of Holocene sediment on the Chukchi-Alaskan margin based on combined diffuse spectral reflectance and quantitative X-Ray Diffraction analysis. *Global and Planetary Change* **68**, 73-84 (2009).

15. Stein, R. *et al.* Towards a better (litho-) stratigraphy and reconstruction of Quaternary paleoenvironment in the Amerasian Basin (Arctic Ocean). *Polarforschung* **79**, 97-121 (2010).

16. Burdige, D. J., Alperin, M. J., Homstead, J. & Martens, C. S. The Role of Benthic Fluxes of Dissolved Organic Carbon in Oceanic and Sedimentary Carbon Cycling. *Geophysical Research Letters* **19**, 1851-1854 (1992).

17. Burdige, D. J. & Komada, T. *Sediment pore waters*. 2nd eds, 536-569 (Academic Press, 2014).

18. Ardyna, M. *et al.* Parameterization of vertical chlorophyll *a* in the Arctic Ocean: impact of the subsurface chlorophyll maximum on regional, seasonal, and annual primary production estimates. *Biogeosciences* **10**, 4383-4404 (2013).

19. Ardyna, M. *et al.* Recent Arctic Ocean sea ice loss triggers novel fall phytoplankton blooms. *Geophysical Research Letters* **41**, 6207-6212 (2014).

Table S1. Summary of sites description and pore water characteristics (mean ± SD).

Item	Unit	S1 (LPC-1a) n=14	S2 (LPC-2) n=5	S3 (LPC-3) n=10	S4 (LPC-4) n=10
Sampling time		Sep.5, 2015	Aug.27, 2015	Aug.30, 2015	Sep.2, 2015
Latitude		73°37'13"N	76°36'10"N	75°32'00"N	76°25'52"N
Longitude		166°25'44"W	161°10'05"W	178°44'04"E	172°40'36"W
Water depth	m	100	2077	715	2240
SMTZ depth	mbsf	~ 4.8	> 3.5	> 4.9	> 10.5
Salinity	psu	33.7 ± 0.5	36.8 ± 1.1	35.8 ± 0.5	35.3 ± 0.6
Sea ice cover		ice-free	partially	partially	partially
DOC	mM	6.6 ± 2.4	4.4 ± 1.9	2.5 ± 0.8	8.4 ± 3.6
a(254)	m^{-1}	211 ± 78	23 ± 17	23 ± 12	12 ± 4
a(350)	m^{-1}	53 ± 18	9 ± 8	11 ± 9	3 ± 2
a(254)*	$L(mgC\text{-}m)^{-1}$	2.7 ± 0.9	0.6 ± 0.5	0.8 ± 0.5	0.1 ± 0.1
C1	RU	34.5 ± 20.4	0.4 ± 0.4	0.7 ± 0.5	0.3 ± 0.2
C2	RU	16.8 ± 8.0	0.3 ± 0.2	0.7 ± 0.5	0.3 ± 0.1
C3	RU	11.9 ± 5.2	1.2 ± 1.8	0.5 ± 0.3	0.2 ± 0.1
C4	RU	16.8 ± 8.7	2.1 ± 1.9	1.6 ± 0.6	2.4 ± 1.0
C1	%	39 ± 9	15 ± 8	18 ± 7	9 ± 5
C2	%	22 ± 3	13 ± 9	19 ± 5	11 ± 4
C3	%	17 ± 4	25 ± 20	14 ± 4	8 ± 3
C4	%	23 ±7	48 ± 16	50 ± 16	72 ± 9
Alkalinity	mM	33 ± 13	3 ± 0	3 ± 0	4 ± 1
NH_4^+	mM	2.3 ± 1.1	0.1 ± 0.0	0.2 ± 0.1	0.1 ± 0.0
PO_4^{3-}	µM	263 ± 70	0.0 ± 0.0	1.2 ± 3.8	3.0 ± 4.7

Table S2. Fractional porosity of surficial sediments.

Depth (mbsf)	S1 (JCP-1a)	S2 (JCP-2)	S3 (JCP-3)	S4 (JCP-4)
0.001	0.95	0.77	0.88	0.93
0.011	0.91	0.74	0.78	0.78
0.021	0.90	0.72	0.76	0.76
0.031	0.89	0.71	0.75	0.73
0.041	0.89	0.70	0.73	0.69
0.051	0.88	0.68	0.72	0.68
0.061	0.88	0.65	0.72	0.63
0.071	0.88	0.63	0.72	0.61
0.081	0.88	0.61	0.71	0.61
0.091	0.88	0.60	0.69	0.60
0.101	0.87	0.59	0.68	0.58
0.111	0.86	0.58	0.67	0.59
0.121	0.84	0.59	0.67	0.61
0.131	0.84	0.62	0.67	0.60
0.141	0.84	0.64	0.67	0.60
0.151	0.83	0.66	0.66	0.59
0.161	0.83	0.67	0.65	0.58
0.171	0.83	0.67	0.64	0.52
0.181	0.83	0.68	0.63	0.55
0.191	0.82	0.69	0.62	0.57
0.201	0.82	0.70	0.62	0.58

Table S3. Coefficients of Pearson's correlation among the DOM variables and water chemistry index (n = 39, $p < 0.01$).

Item	DOC	*a*(254)	*a*(254)*	C1	C2	C3	C4	%C1	%C2	%C3	%C4	Salinity	Alkalinity	NH_4^+	PO_4^{3-}	SO_4^{2-}
DOC																
a(254)			0.94	0.97	0.99	0.99	0.97	0.91			-0.76	-0.80	0.99	0.98	0.96	-0.97
a(254)*				0.87	0.90	0.93	0.86	0.87			-0.81	-0.74	0.89	0.87	0.91	-0.86
C1				0.99	0.99	0.96	0.99	0.88			-0.68	-0.75	0.98	0.98	0.88	-0.97
C2						0.98	0.98	0.91			-0.73	-0.78	0.99	0.99	0.93	-0.98
C3							0.95	0.88			-0.75	-0.78	0.98	0.96	0.95	-0.96
C4								0.85			-0.64	-0.76	0.97	0.97	0.88	-0.96
%C1									0.72		-0.88		0.90	0.90	0.87	-0.88
%C2											-0.82					
%C3																
%C4													-0.73	-0.73	-0.77	
Salinity													-0.80	-0.78	-0.80	0.82
Alkalinity														0.99	0.94	-0.99
NH_4^+															0.91	-0.98
PO_4^{3-}																-0.92
SO_4^{2-}																

Table S4: Depth-integrated POCSR rates for the four investigated sites.

	Unit	S1	S2	S3	S4
Core length	m	6.8	3.54	4.92	10.5
Maximum rate	μmol C $cm^{-2}yr^{-1}$	30.7	1.5	4.9	0.4
Minimum rate	μmol C $cm^{-2}yr^{-1}$	13.4	0.9	2.2	0.2
Average rate	μmol C $cm^{-2}yr^{-1}$	22.05	1.2	3.55	0.3

Table S5. Estimation of benthic flux of CDOM and FDOM (Diffusion flux J = sediment porosity x diffusion coefficient x concentration gradient, i.e., $J = \phi_o Ds(\partial C/\partial z)_o$. Positive values stand for the efflux from sediments to overlying water and vice versa. RU: Raman Unit.

Variables	Unit	S1	S2	S3	S4
a(350)	$(m^{-1}$ L) m^{-2} yr^{-1}	24.7-58.7	27.6-65.7	1.4-3.4	-(12.5-29.7)
C1	(RU L) m^{-2} yr^{-1}	7.7-18.3	0	0	0
C2	(RU L) m^{-2} yr^{-1}	9.3-22.1	0	0	0
C3	(RU L) m^{-2} yr^{-1}	10.4-24.6	6.7-16.0	0	0
C4	(RU L) m^{-2} yr^{-1}	19.6-46.4	0.2-0.6	-(14.7-34.9)	-(0.1-0.3)

Table S6. Summary of DOM, nutrients, and ion parameters.

				CDOM:		FDOM:								Nutrients:		Ion:
Site	Depth (mbsf)	Salinity (psu)	DOC (mM)	*a*(254) (m^{-1})	*a*(350) (m^{-1})	C1 (RU)	C2 (RU)	C3 (RU)	C4 (RU)	C1 (%)	C2 (%)	C3 (%)	C4 (%)	NH_4^+ (mM)	PO_4^{3-} (μM)	Alkalinity (mM)
S1:																
1	0.2	34.0	4.7	36.0	12.2	0.7	0.9	1.0	2.2	14.9	19.0	20.8	45.3	0.2	89.6	4.1
2	0.7	35.0	4.9	78.5	21.3	3.4	3.8	3.5	3.4	24.4	27.2	24.5	23.9	1.0	175.5	11.9
3	1.2	34.0	5.0	141.3	38.2	9.7	7.9	6.8	5.7	32.2	26.1	22.6	19.0	1.5	245.4	20.1
4	1.5	34.0	4.6	165.6	44.8	14.1	10.1	8.5	10.0	33.0	23.6	20.0	23.5	1.1	285.2	23.7
5	2.29	34.0	5.1	199.6	52.8	24.1	14.9	12.0	11.8	38.3	23.8	19.1	18.8	1.4	294.5	29.9
6	2.79	33.5	6.9	233.2	63.2	37.2	19.7	16.0	17.0	41.3	21.9	17.8	19.0	2.3	316.7	35.0
7	3.29	33.5	6.9	242.0	61.9	35.3	18.0	13.9	16.3	42.3	21.5	16.6	19.6	2.7	315.4	39.2
8	3.79	33.5	6.6	266.5	68.0	48.9	22.5	17.2	22.6	43.9	20.3	15.5	20.3	2.8	255.1	41.2
9	4.29	33.5	6.6	274.2	69.8	50.1	21.6	15.6	27.1	43.8	18.9	13.6	23.7	3.1	360.4	43.3
10	4.79	33.5	6.5	286.2	73.5	62.3	25.7	17.9	24.5	47.8	19.7	13.7	18.8	3.4	334.5	43.3
11	5.34	33.5	7.2	277.1	67.6	55.7	24.4	15.9	24.0	46.4	20.4	13.2	20.0	3.2	282.9	43.3
12	5.84	33.5	14.1	245.9	57.7	45.3	21.7	12.8	21.2	44.9	21.5	12.6	21.0	3.3	271.7	43.3
13	6.34	33.5	6.6	257.9	60.4	51.7	23.4	13.2	27.4	44.7	20.2	11.4	23.7	3.3	248.8	43.3
14	6.84	33.0	6.8	245.6	57.6	44.0	20.7	12.8	21.3	44.5	20.9	13.0	21.5	3.0	205.3	43.3
S2:																
1	0.1	35.5	2.5	41.1	17.3	0.0	0.0	4.3	3.3	0.0	0.0	56.4	43.6	0.1	0.0	2.6
2	0.6	36.0	2.8	10.1	2.2	0.2	0.3	0.5	0.8	12.5	17.0	27.9	42.5	0.0	0.0	3.6
3	1.54	37.9	7.1	8.2	2.5	0.4	0.3	0.3	0.8	20.3	18.8	17.3	43.6	0.0	0.0	3.6
4	2.54	37.9	5.5	12.1	4.2	0.6	0.5	0.5	0.8	25.1	20.0	20.2	34.7	0.1	0.0	3.6
5	3.54	36.9	3.9	41.4	19.1	0.9	0.4	0.2	4.8	14.9	7.0	3.0	75.2	0.1	0.0	3.6
S3:																
1	0.1	36.0	2.5	6.1	1.9	0.0	0.1	0.1	1.0	0.3	7.9	7.7	84.1	0.0	0.0	2.6
2	0.6	36.0	2.4	19.4	10.5	0.5	0.4	0.3	2.3	14.7	11.7	7.8	65.7	0.0	0.0	3.1

3	1.1	36.0	1.2	21.0	10.2	0.6	0.6	0.5	1.0	22.3	21.3	18.5	37.9	0.0	0.0	3.1
4	1.6	36.0	2.4	26.0	14.5	0.6	0.6	0.5	1.7	17.8	18.2	15.1	49.0	0.1	0.0	3.1
5	2.1	35.0	2.1	42.2	32.6	0.8	0.7	0.6	1.4	22.2	21.3	16.5	39.9	0.1	11.9	3.1
6	2.42	36.0	2.4	20.1	9.2	0.6	0.6	0.4	1.5	18.3	19.6	13.9	48.2	0.2	0.0	3.1
7	2.92	35.0	3.3	14.5	4.0	0.4	0.5	0.3	1.4	15.8	19.6	12.4	52.2	0.2	0.0	3.6
8	3.92	35.5	1.6	10.7	2.0	0.5	0.5	0.3	0.9	23.1	23.8	12.9	40.2	0.3	0.0	3.6
9	4.42	36.5	3.6	25.1	6.6	1.0	1.0	0.6	2.6	18.9	19.3	11.9	49.9	0.4	0.0	3.6
10	4.92	36.0	3.7	40.5	18.7	1.9	1.8	1.3	2.1	26.6	25.9	18.3	29.3	0.5	0.0	4.1
S4:																
1	0.25	36.0	9.4	7.2	2.5	0.0	0.1	0.1	2.8	1.1	4.4	3.8	90.7	0.0	4.5	2.6
2	0.75	35.0	7.0	6.2	0.8	0.0	0.2	0.2	1.8	1.5	8.5	10.6	79.4	0.0	0.0	3.1
3	1.25	36.0	4.0	12.4	3.8	0.2	0.3	0.2	1.9	6.0	11.4	9.0	73.6	0.1	0.0	3.6
4	1.75	35.0	12.5	17.8	5.0	0.7	0.3	0.1	4.9	11.7	5.6	1.2	81.5	0.0	0.0	3.6
5	3.07	36.0	7.5	17.6	5.8	0.5	0.5	0.4	2.0	15.5	14.1	11.4	59.0	0.0	3.1	3.6
6	3.57	35.0	3.0	16.2	5.3	0.6	0.5	0.3	2.7	15.2	11.8	8.0	65.0	0.0	0.0	3.6
7	4.07	35.0	8.0	12.2	3.6	0.4	0.4	0.3	1.9	13.3	13.0	9.9	63.7	0.0	0.0	3.6
8	4.57	36.0	11.4	9.6	1.5	0.3	0.3	0.2	1.9	10.8	12.3	8.5	68.4	0.1	0.0	4.1
9	8.5	35.0	7.0	7.2	1.5	0.2	0.3	0.2	2.5	6.9	9.6	7.3	76.2	0.1	10.7	5.7
10	10.5	34.5	14.4	10.7	2.1	0.4	0.5	0.3	1.9	12.5	15.3	10.8	61.5	0.1	12.0	6.2

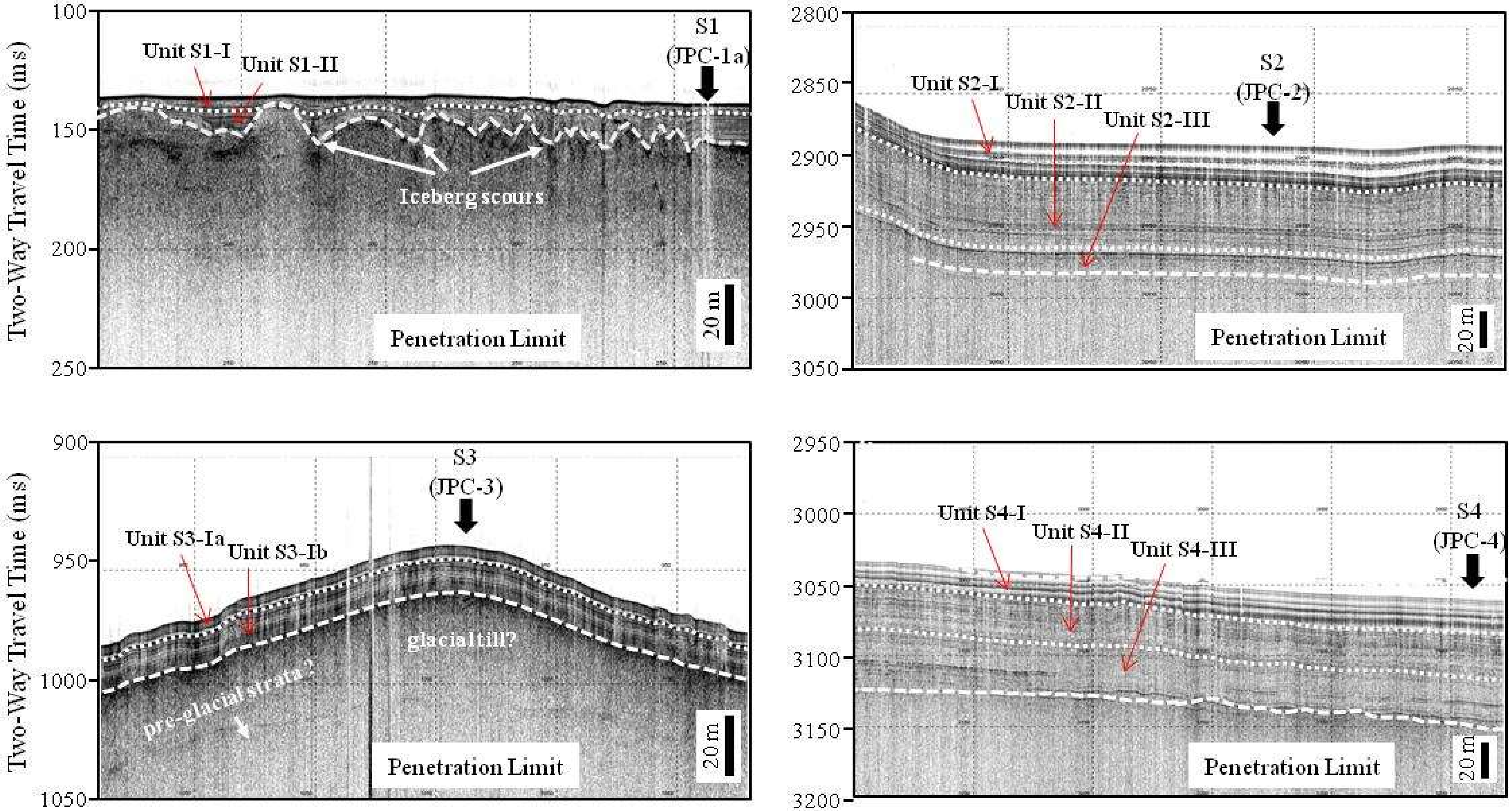

Fig. S1. CHIRP sub-bottom profiles crossing the sampling sites for pore waters in the Arctic Ocean.

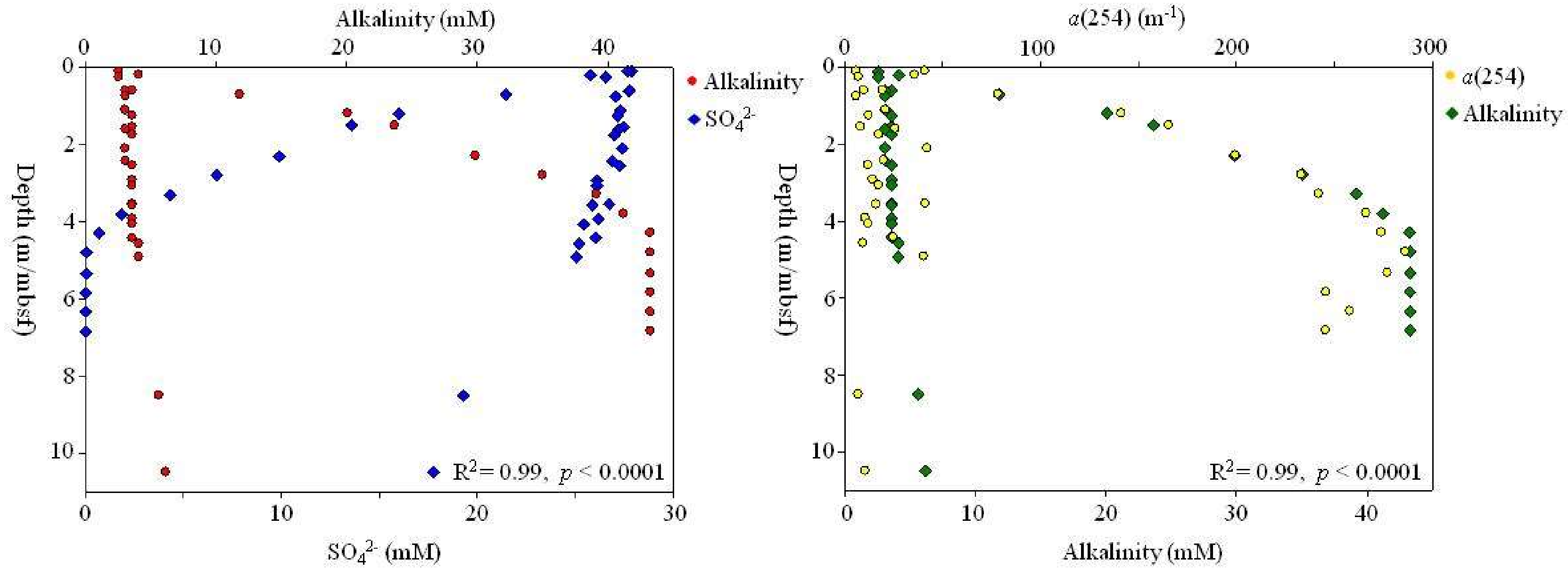

Fig. S2. Positive relationships between CDOM and alkalinity (left). Inverse relationships between alkalinity and sulfate (right).

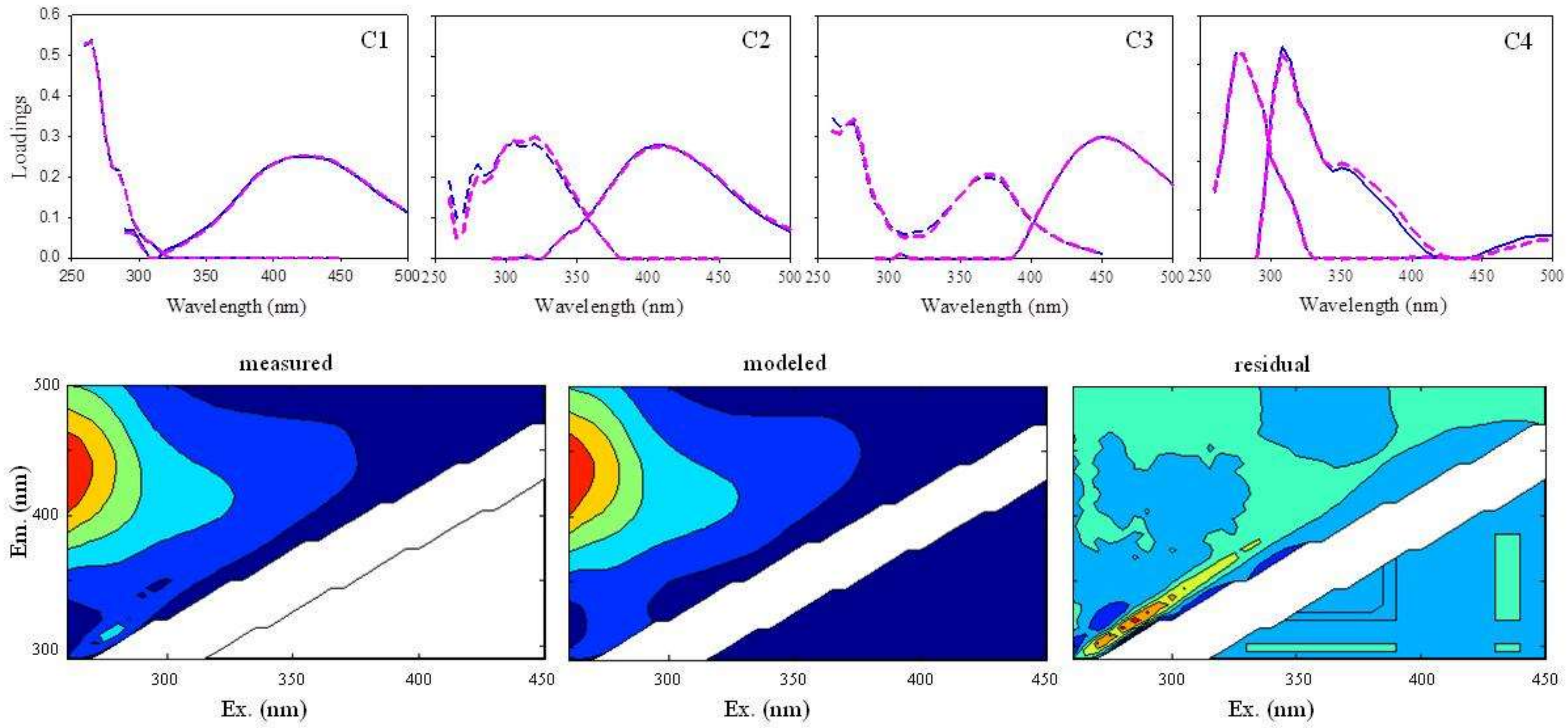

Fig. S3. Split-half validation of the identified four EEM-PARAFAC components and an example of the measured, modeled, and residual EEM.

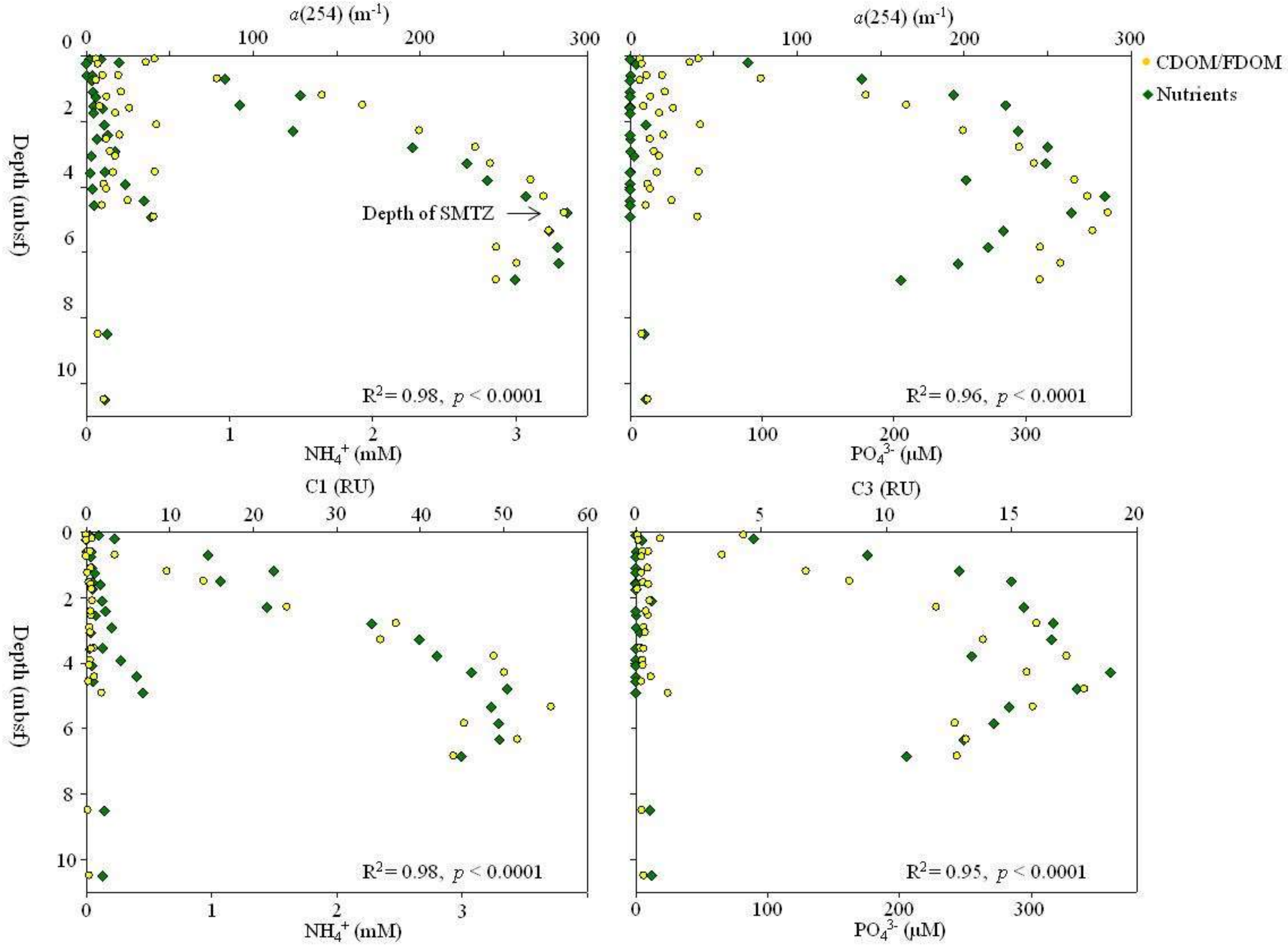

258

259 Fig. S4. Pore water downcore profiles between CDOM as well as FDOM and nutrients of NH_4^+ and PO_4^{3-}.

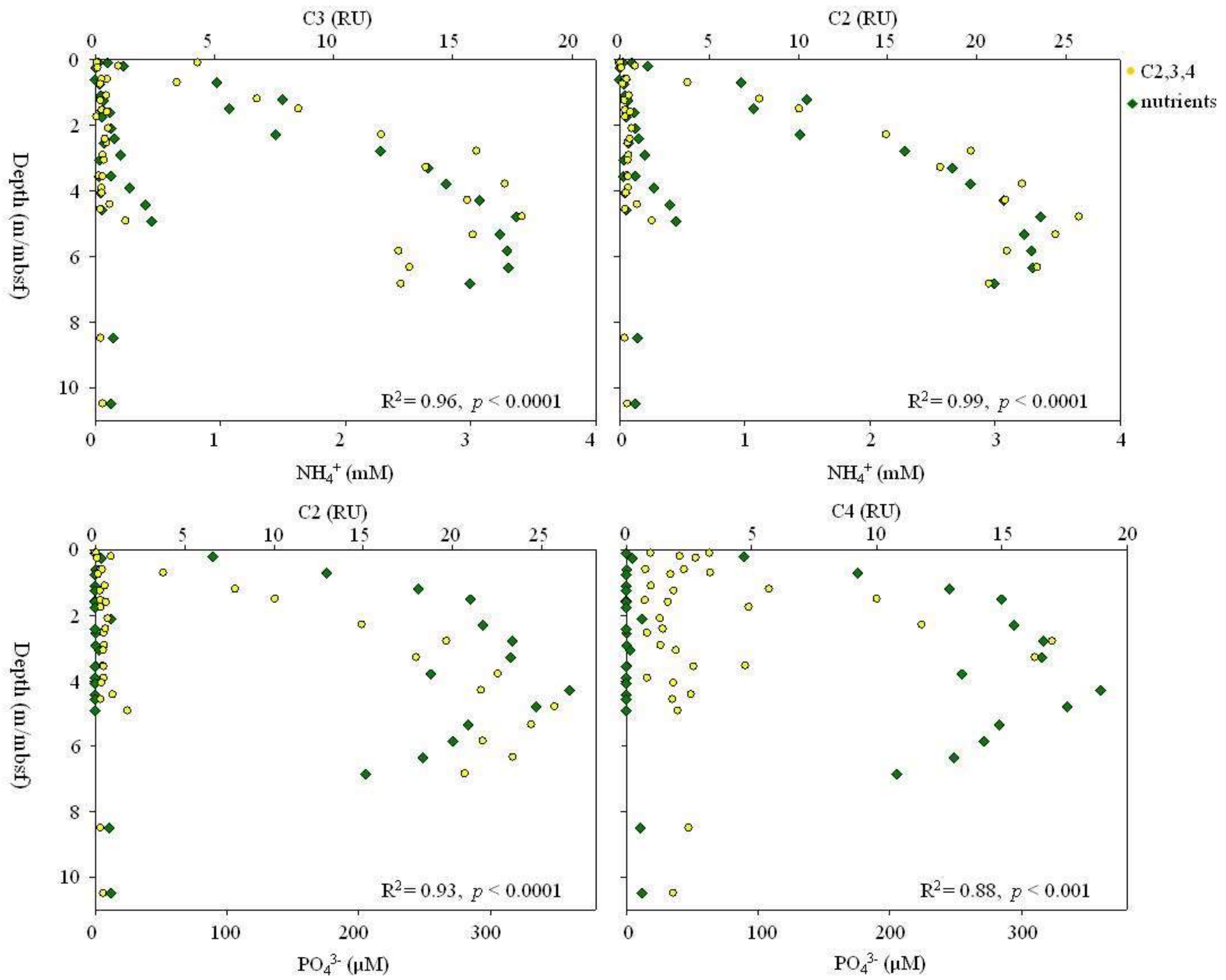

260

261 Fig. S5. Pore water downcore profiles between CDOM and FDOM with nutrients (NH_4^+ and PO_4^{3-}).

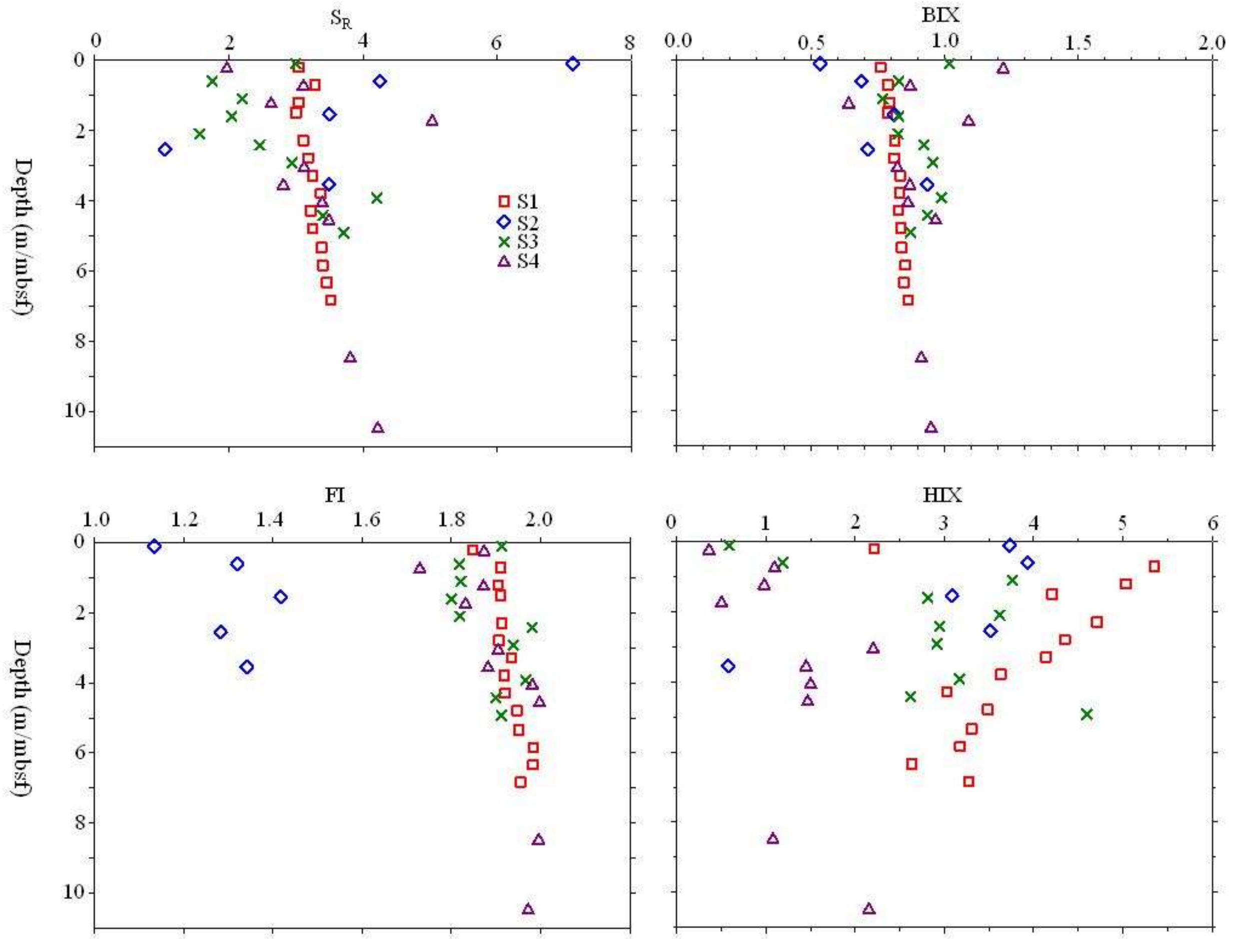

Fig. S6. Downcore profiles of selected DOM optical indices in the Arctic pore waters.

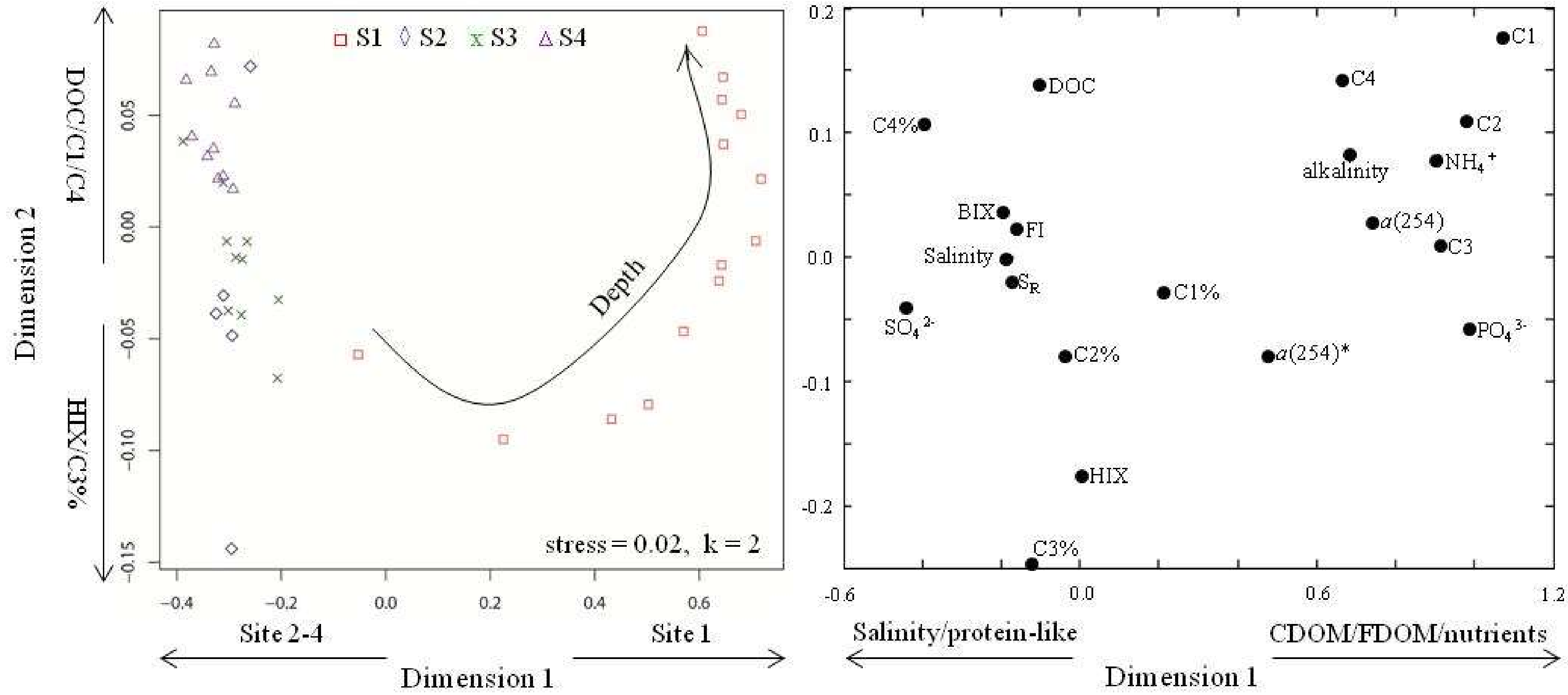

Fig. S7. Non-metric multidimensional scaling (NMS) of the pore water samples based on DOM optical data and water chemistry data. Ordinations based on Bray–Curtis distance.
